# Post-overdose interventions triggered by calling 911: Centering the perspectives of people who use drugs (PWUDs)

**DOI:** 10.1371/journal.pone.0223823

**Published:** 2019-10-17

**Authors:** Karla D. Wagner, Robert W. Harding, Richard Kelley, Brian Labus, Silvia R. Verdugo, Elizabeth Copulsky, Jeanette M. Bowles, Maria Luisa Mittal, Peter J. Davidson

**Affiliations:** 1 School of Community Health Sciences, University of Nevada, Reno, Nevada, United States of America; 2 Nevada Center for Applied Research, University of Nevada, Reno, Nevada, United States of America; 3 School of Public Health, University of Nevada, Las Vegas, Nevada, United States of America; 4 FirstWatch, Inc., Carlsbad, California, United States of America; 5 Division of Infectious Disease and Global Public Health, Department of Medicine, University of California, San Diego, California, United States of America; Douglas Mental Health University Institute, CANADA

## Abstract

**Background:**

Opioid overdose deaths have increased exponentially in the United States. Bystander response to opioid overdose ideally involves administering naloxone, providing rescue breathing, and calling 911 to summon emergency medical assistance. Recently in the US, public health and public safety agencies have begun seeking to use 911 calls as a method to identify and deliver post-overdose interventions to opioid overdose patients. Little is known about the opinions of PWUDs about the barriers, benefits, or potential harms of post-overdose interventions linked to the 911 system. We sought to understand the perspectives of PWUDs about a method for using 911 data to identify opioid overdose cases and trigger a post-overdose intervention.

**Methods and findings:**

We conducted three focus groups with 11 PWUDs in 2018. Results are organized into 4 categories: willingness to call 911 (benefits and risks of calling), thoughts about a technique to identify opioid overdoses in 911 data (benefits and concerns), thoughts about the proposed post-overdose intervention (benefits and concerns), and recommendations for developing an ideal post-overdose intervention. For most participants, calling 911 was synonymous with “calling the police” and law enforcement-related fears were pervasive, limiting willingness to engage with the 911 system. The technique to identify opioid overdoses and the proposed post-overdose intervention were identified as potentially lifesaving, but the benefits were balanced by concerns related to law enforcement involvement, intervention timing, and risks to privacy/reputation. Nearly universally, participants wished for a way to summon emergency medical assistance without triggering a law enforcement response.

**Conclusions:**

The fact that the 911 system in the US inextricably links emergency medical assistance with law enforcement response inherently problematizes calling 911 for PWUDs, and has implications for surveillance and intervention. It is imperative to center the perspectives of PWUDs when designing and implementing interventions that rely on the 911 system for activation.

## Introduction

Opioid overdose deaths have risen exponentially in the United States (US)[1]. In 2017, over 70,000 people died from drug overdose in the US [2]. This number translates to an age-adjusted rate of 21.7/100,000, representing a 9.6% increase compared to 2016 [2]. Of those drug-related deaths, deaths involving synthetic opioids (other than methadone), increased by 45% from 2016 to 2017 [2]. Polypharmacy is also a major contributor to overdose deaths, especially consumption of opioids in combination with benzodiazepines and, increasingly, stimulant drugs such as methamphetamine and cocaine [3].

While the number and rates of opioid overdose have increased dramatically in recent years, drug overdose has been identified as a leading cause of preventable death among people who use drugs (PWUDs) for at least two decades [4], and interventions to train PWUDs in opioid overdose prevention/response using take-home naloxone have existed in the US since the late 1990’s [5]. While the initial scale up was slow across the US, by 2014 there were 644 sites providing take-home naloxone to PWUDs [6]. These programs typically train bystanders to respond to opioid overdose by administering naloxone, providing rescue breathing and/or CPR, and summoning emergency medical assistance by calling 911. Summoning emergency medical services can be an important step for bystanders. Because naloxone is an opioid antagonist with specific affinity for the opioid receptors, a polypharmacy overdose caused by multiple substances may not be reliably reversed with naloxone administration alone. While rare, other sequalae (e.g., pulmonary edema subsequent to opioid use or acetaminophen toxicity) are possible and many opioid overdose patients could benefit from additional supportive care for optimal recovery [7].

In the US, 911 is a universal telephone number operating under local governance that routes calls to a system of call centers, ultimately resulting in the dispatch of emergency personnel. Typically, calls are received by a public safety answering point (PSAP), which identifies the nature of the emergency and either dispatches responders immediately or transfers the call to a more specialized secondary PSAP [8]. Medical emergency calls may be handled by the primary PSAPs, which are usually operated by public safety agencies, or may be routed to call centers with dedicated medical dispatch. While some variability exists in terms of how 911 calls are handled, most dispatch centers use a formalized protocol for asking questions to determine the nature of the emergency and appropriate level of response, dispatch emergency responders, and record data about the call.

For many people who do not use drugs, calling 911 for a medical emergency is a relatively uncomplicated act that represents activating the emergency response system and summoning emergency medical responders to provide lifesaving medical assistance. However, for non-majority populations in the US, including people of color, undocumented immigrants, people living in low income neighborhoods, youth, and PWUDs, calling 911 may be fraught with fear that it might act to summon law enforcement or have other risky consequences (e.g., [9, 10]). In fact, global studies among PWUDs have reported rates of calling for medical assistance between 21% and 63% [11–16].

Multiple factors influence the likelihood of calling 911 for an overdose emergency, including history of experiencing or witnessing overdose [12], social norms or social influence [12], source of information about opioid overdose prevention [14], and location of drug use/overdose [13, 17]. In addition to these individual, social, and structural predictors, one of the most critical influences on whether PWUD summon emergency medical assistance is the legal/policy environment [18]. In the US, where drug use is severely criminalized, research has consistently identified fear of law enforcement as a significant deterrent to calling 911 [12, 14, 19]. PWUDs fear arrest for drug-related charges, but also for charges related to homicide (if the overdose victim dies and those at the scene were involved in some way with providing the drugs used by the decedent), violation of parole/probation, outstanding warrants, and trespassing [20]. These fears are not unfounded, since PWUDs do report that law enforcement officers attend medical emergency calls and individuals at the scene can be arrested as a consequence. For example, participants in an opioid overdose prevention program in Los Angeles, California, USA reported that law enforcement officers responded to 67% of events, and someone was arrested in 14% of events [15]. A community-level study in New York City demonstrated a positive association between the rate of misdemeanor arrests (an indicator of policing) and accidental drug overdose mortality [21]. 911 Good Samaritan laws are designed to reduce these concerns by providing protections against some offenses when someone calls 911 in good faith to summon emergency assistance, but research since their passage suggests that the laws often do not provide sufficient protection to mitigate fears [22].

The epidemic scale of opioid overdose deaths in the US has necessitated the rapid development and scaleup of innovative public health responses to reduce death rates. In many communities, public health, public safety, and social service agencies are exploring the possibility of using the 911 emergency response system as a mechanism for monitoring trends in opioid overdoses and initiating intervention efforts. For example, “post-overdose outreach” interventions represent novel collaborations between public health and public safety agencies to provide outreach and engagement services to people who use opioids and/or their social networks once they come to the attention of the system via an emergency response [23]. Program models include post-overdose outreach to the overdose victim’s residence (either by police, clinicians, or a multidisciplinary team), referrals to services for the overdose victim and their social network, or encouragement that the overdose victims visit a fixed community-based site for services. Other law enforcement-based models include referrals initiated by officers at the scene [24], or via a program that encourages people who use opioids to seek help at the police station [25]. A critical assumption underlying these interventions is that opioid overdose patients will come to the attention of the public health and public safety agencies via a request for emergency medical services (i.e., by calling 911). However, given the substantial literature that describes highly salient and severe risks perceived by PWUDs when considering a call to 911, this assumption requires further interrogation.

The current study was initially undertaken to provide pilot data to inform a post-overdose outreach model similar to those mentioned above. We aimed to investigate the feasibility of using a machine-learning algorithm to identify opioid overdoses in data from the 911 emergency medical dispatch system, which could then be used to trigger a post-overdose outreach intervention. However, given the well-established findings regarding the deterrents to calling 911, we first sought to investigate the ethics and acceptability of such an initiative with PWUDs. In the current study we report on findings from focus groups with PWUDs designed to elicit their perspectives on post-overdose interventions triggered by 911 calls. We present these findings to advance a patient-centered perspective on the development of interventions that affect the health and wellbeing of PWUDs [26].

## Materials and methods

### Setting & legal environment

The study took place in Reno, Nevada. In 2015, the Nevada Legislature passed State Bill 459, Nevada’s Good Samaritan Drug Overdose Act, which amended multiple sections of Title 40 of the Nevada Revised Statue (Good Samaritan Drug Overdose Act, 2015). This law expanded access to naloxone through multiple mechanisms and enacted liability protections for individuals involved in naloxone distribution/prescribing. The law also provided 911 Good Samaritan protections that protect people who call 911 in the event of an overdose or who are the subject of such a call from arrest, charge, prosecution, conviction, or asset forfeiture for use or possession of small amounts of drugs (unless it is for the purpose of sale), possession of drug paraphernalia, or violation of restraining orders or probation or parole. The law explicitly does not prohibit the government from taking action related to the abuse or neglect of a child.

### Recruitment & data collection

The Institutional Review Board (IRB) at the University of Nevada, Reno approved all study activities under protocol #1024876. Respondents provided verbal informed consent using a Consent Information Script, which described the study and the risks/benefits of participation. Because a signature would have been the only identifying information provided by participants, the study was granted a waiver of documentation of consent by the University of Nevada, Reno IRB.

We conducted three focus groups over the course of one week in 2018. Participants were recruited through the distribution of flyers at community-based organizations that provide services for PWUDs, including substance use disorder treatment centers and syringe services programs, and via word of mouth, resulting in three groups of participants who came from different locations in town and different networks of PWUDs. Criteria for eligibility included being over 18 years old and a current opioid user. Recruitment flyers described the study as seeking to hear the opinions of people who use opioids about a new intervention to reduce opioid overdoses. Focus groups were conducted at a university-leased research field site, located in a nondescript building accessible by foot, bike, bus, or car. Participants received $20 for their time and food was provided at the focus group. After the focus groups were complete, participants were offered the opportunity to participate in a brief overdose education session and were provided naloxone by a community-based organization that partnered with the study.

Focus groups were facilitated by the first author, a behavioral scientist with 20 years of experience in qualitative data collection, and were attended by 3 additional authors who served as observers, note takers, and co-facilitators as needed. The loosely-structured interview guide began with a scripted description of the proposed intervention: “The idea for the project would be to identify opioid overdoses based on the data that come in on a call to 911, then send a counselor or a peer educator to the scene to provide naloxone, training in overdose risk reduction techniques, and/or connection to services like methadone, buprenorphine, or syringe services.” The interventionist was subsequently described as a peer recovery support specialist (i.e., someone with lived experience of substance use who is trained in recovery support). Then, participants were asked what they think of the idea and what benefits/harms might result from such a program. Throughout the focus groups, the technique and intervention were described in increasingly more detail, with additional examples of how they could be implemented and the potential benefits and harms. For example, we told participants that the machine-learning technique would use data from the 911 call to identify overdose cases, even if the caller did not explicitly state that the call was for an overdose (e.g., by using other contextual information from the call). We also asked participants about how knowledge of such a program would affect their willingness to call 911. The focus groups were digitally recorded and facilitators took notes to record impressions. Audio recordings were transcribed verbatim, and reviewed to ensure any identifiable information was redacted prior to analysis.

### Analysis

The first author conducted the qualitative analysis using a thematic approach that relied on *a priori* categories and emergent themes [27, 28]. First, transcripts were loaded into the Atlas.ti software program for management. Transcripts were read in their entirety and memos were used to document initial impressions. Codes were developed iteratively, based on the initial reading of the transcripts, and were grouped into higher and lower order concepts, based on our *a priori* questions about participants’ impressions of the proposed technique and intervention. Codes were applied systematically to the transcripts for each focus group. Then, the coded segments of the transcripts were output and read a final time, during which additional axial codes were applied. Findings were shared with authors 2, 5, 6, 7, 8, and 9 and interpretations were solidified through reflection, discussion, and revision until consensus was achieved.

## Results

Three focus groups were attended by 11 participants (Focus group [FG] 1: 2 women; FG2: 1 woman, 5 men; FG3: 1 woman, 2 men) over the course of one week in November, 2018. Focus groups lasted between 40 and 55 minutes in duration. We present findings related to four *a priori* categories that formed the basis for our inquiry: (1) willingness to call 911 and the fears and meanings associated with that act, (2) thoughts about the development of a machine-learning technique to identify opioid overdoses in 911 data, (3) thoughts about the subsequent intervention that could be deployed, and (4) recommendations for an ideal post-overdose intervention. Within each category, we present emergent themes and supporting narratives from the focus group transcripts. The categories and themes are summarized in Table 1.

**Table 1:** *A priori* categories and emergent themes related to PWUDs’ perspectives on a post-overdose outreach intervention triggered by calling 911.

**Category 1: Willingness to call 911**	
Benefits of calling:	Risks of calling:
- Save a life	- Fear of CPS/losing children
	- Fear of police
	- Fear of arrest and/or incarceration
	- Impact on privacy/reputation
**Category 2: Thoughts about technique to identify overdoses in 911 data**	
Benefits:	Concerns:
- Shorten time to appropriate care	- Inaccuracy, leading to misdiagnosis or delayed treatment
- Save a life	- Privacy, violation of choice not to disclose
**Category 3: Thoughts about a post-overdose intervention**	
Benefits:	Concerns:
- Peer support specialist could empathize with patient	- Privacy, violation of confidentiality
	- Timing of intervention is suboptimal because of precipitated withdrawal
	- Disincentivize transport to hospital
**Category 4: Recommendations for an ideal post-overdose intervention**	
- Active follow-up (not just referrals) - Flexible intervention, allow people to talk about their “real problems” not just substance use disorder treatment - Create alternative number that can summon emergency medical assistance without linking to law enforcement	

### Willingness to call 911

Nearly universally, respondents described hesitance to call 911 in the event of an overdose. For example, one respondent said,

I feel threatened every time if I have to call for assistance, because of cops….Now, if a friend is overdosing, “Oh shit. I’ve got to call 911. Cops are going to come too. Oh fuck. They’re going to start harassing me again.”

The exchange below between the interviewer (I) and two female respondents (R1 and R2) describes the multifaceted nature of people’s concerns, and highlights the additional fears faced by people with children:

I: So, the question is, ‘How worried are you right now, about…right now how worried would you be about calling 9-1-1 for somebody that was overdosing’?

R2: As worried as a person could be. I would do everything in my power not to call 9-1-1.

R1: And I’m opposite. I’ve always called because, like I said, if it comes down to it and I have to go to prison, I trust in God that … at least I know that I saved someone’s life. Like, I will take the repercussions, I don’t hesitate.

I: And you both talked about this a little bit, but the question says, ‘What are you worried about exactly’?

R2: Um, my children [laughter].

I: Mhm.

R1: And [I] think me, that’s why I don’t hesitate so much anymore, ‘cause I’ve already lost my children. I don’t want to lose my dog, but…you know, she’s still just a dog. It’s a lot different from losing a child so…

R2: So yeah, that’s my number one concern. Also my, my reputation, I don’t want anyone to know anything about me, so I could… there’s a good possibility I could know a first responder or know somebody from, you know.

I: Mmmm. The person who shows up? The people who show up are people that you might know?

R2: Sure.

I: And then they would know things about you?

R2: Yeah, or going, or ending up in the hospital or something, that I could know an RN or I could know the social worker or any number of people.

The passage above highlights one respondent’s fear that a call to 911 could involve her with Child Protective Services (CPS) and lead to the loss of her children. Later, she goes on to say, “I fear CPS so much, because my children are my world, you know? And…that makes me want to at least steer clear of law enforcement regardless of…you know…it’s a really, really, really, really big deal.” The second respondent in the passage above reveals that she has already lost children, though the circumstances leading to that loss are not discussed. The ultimate consequence for her would be going to prison, though she says she would be willing to face that consequence in the interest of saving a life. Later in the conversation, this respondent talked about fear of facing murder charges if she were at the scene of a fatal overdose. The first respondent also highlights that a call to 911 could threaten one’s privacy and impact one’s reputation. This fear was echoed by a male respondent in another focus group, who described an experience in which he had encountered individuals through both the emergency medical and law enforcement systems (e.g., a law enforcement officer who was also a volunteer paramedic), and believed that information about his drug use learned through the clinical encounter was subsequently used against him during a law enforcement investigation.

For many, “calling 911” was synonymous with “calling the police”. For example, when asked by the interviewer (I) about what would make him more or less willing to call 911 in the event of an opioid overdose, one participant (R) explained through a hypothetical example that calling 911 would be a last resort only if the administration naloxone did not work,

R: Personally…this is make-believe, pretend…this would only be if the naloxone that I already administered myself wasn’t working, that I would be having *to call the police*. [emphasis added]

I: You do what you can first, and then…?

R: “Hell yeah. All things. I don’t *call the police* ever, for any reason, unless I have exhausted every means.” [emphasis added]

In this passage we see that even though the interviewer was asking about willingness to call 911, the respondent answered by talking about “calling the police”. Another respondent described the events following his own opioid overdose, including his observation of the arrests of his friends and his perceived risk of arrest himself,

Yeah…I overdosed, they *call[ed] the cops for me*. [emphasis added] Yet, when I came to after they did Narcan on me, the cops were arresting my friend because they were high too, and they had dope on them, but they went to jail. I was in the hospital and I was next to go to jail, but I snuck out. And they were waiting. They were. I seen them and I overheard them and everything.

The semantic substitution of “police” and “cops” for “911” in these quotes reveals how deeply connected the 911 and law enforcement systems are in the minds of these respondents. To many, there is no difference between calling 911 to summon emergency medical assistance and calling 911 to summon law enforcement. In fact, this observation may be accurate in many communities, where the primary PSAP call center may be operated by a public safety agency [8].

### Thoughts about a technique to identify opioid overdoses in 911 data

We asked participants to share their opinions about our idea to develop a machine-learning technique that would use existing data in the 911 dispatch system to identify opioid overdoses. If it worked, this technique would have the potential to use data from the call to identify opioid overdose cases even if the caller did not explicitly state that the medical emergency was for an overdose. Respondents perceived both benefits and concerns related to such a technique. The primary benefit was that respondents believed it could help shorten the time to appropriate care for someone dying of an opioid overdose. This assessment was tied to their understanding of the mechanisms of opioid overdose–specifically, that the respiratory depression associated with opioid overdose must be reversed quickly:

R: If it can save somebody faster, I mean, that’s good, right? I mean…sometimes it’s just minutes without oxygen can cause brain damage.

R: All I can really think of is that it can improve… is that you’re able to realize more quickly that it is an overdose and treat it appropriately.

Concerns related to the technique focused on two domains: inaccuracy and privacy. Some respondents expressed concerns that if the technique were inaccurate, it could contribute to misdiagnosis and/or delayed treatment. For example, one respondent said,

R: Well I guess if they’re focused specifically, if they go into it specifically focused on, with it in their head that this is an overdose, then it might delay them from taking the measures that they need if it was something else. I mean they only have seconds to save lives or save a life, you know?

I: Oh, like if it’s wrong—Then the paramedics go in with an idea about what it is—

R: Exactly and it may delay them a minute or two [cross talk]. So that could be one downfall, but then the upside is that it could also save a life if, you know, people are being honest enough to say, you know, they go in thinking it’s an overdose and it is, they are able to immediately…they go less time without the lack of oxygen, which can cause brain damage. So, it can work both ways, you know.

Privacy concerns were also raised. Some participants believed that some callers have reasons for not disclosing the nature of the call, and that a technique like this would override that choice. This could be particularly problematic for people on parole/probation or other community supervision, if a call to 911 signaled that there was drug use occurring at their house. However, most participants balanced that concern with the potential benefits, as illustrated in the quote above. Some respondents also identified a sense of inevitability regarding the discovery of the true nature of the call,

As soon as they get there, they’re [going] to know anyway. It doesn’t matter what you say…If I say it’s an overdose [or] you get the idea based on this [technique] that it’s an overdose, the end result is the same. It’s an overdose. So, they say it’s an overdose and based on that, you’re going to send somebody to help or whatever, or you get the idea it’s an overdose [based on the technique], you’re going to send somebody. The end result is the same.

### Thoughts about a post-intervention intervention initiated by a 911 call

The machine-learning technique was proposed as a way to identify opioid overdoses in the 911 data, which would allow for the deployment of a peer recovery support specialist or other interventionist to the scene. Respondents perceived both benefits and concerns related to this idea. In general, they thought it was a good idea to send a peer recovery support specialist (i.e., someone with lived experience of substance use who is trained in recovery support), who would know what the patient was going through. This would generate empathy and connection between the patient and the interventionist. Some had seen other similar models, such as mobile crisis intervention teams, and perceived that this kind of intervention might work.

Two primary concerns about the intervention were raised. First, similar to their concerns about the technique to identify opioid overdoses in the 911 dispatch data, participants were concerned about risks to privacy. For some, having an interventionist come to their house would feel invasive, while for others they worried that if the interventionist were known as someone who comes for opioid overdoses, this could compromise confidentiality. Second, some respondents thought that having someone arrive immediately after the overdose was treated was not optimal timing,

I think it’s a good idea that they would have the education to know what type of people they are dealing with and to be more prepared, but I think trying to give a person who is just waking up from an overdose a bunch of information as soon as they wake up is pretty much senseless.

The concern about timing was mostly related to the anticipation that the patient would be experiencing withdrawal symptoms from the opioid overdose reversal, and therefore would not be able to retain or process the information they were given. Others suggested that the benefit of the intervention might be more for the friends or family members or other bystanders, since the patient would likely be transported to the hospital and wouldn’t be able to receive the intervention. One respondent thought that a post-overdose intervention provided on-scene might disincentivize a patient from going to the hospital, which could compromise their health.

### Recommendations for developing a patient-centered intervention

In terms of intervention content, respondents stressed that simply providing referrals is not enough. For some, this sentiment was motivated by experiences in which they hit dead ends when trying to pursue referrals they had been given, leading to the suggestion that there also be active follow-up with patients after the initial intervention and referrals. Others emphasized that the intervention should be flexible enough to allow people to talk about their “real problems”. That is, the intervention should be responsive to and centered on patients’ needs and not exclusively focused on substance abuse treatment.

In all three focus groups, respondents spontaneously generated the same solution to their concerns regarding the risks of calling 911 and the perceived risks of the proposed intervention: Create an alternative number that could be used to summon emergency medical assistance without being connected to the law enforcement dispatch system. For some, this idea was derived from past experiences. For example, one respondent recalled a time when a bystander called directly to the hospital to summon an ambulance for a medical emergency. For others, the idea was generated spontaneously after consideration of the potential risks and benefits of the technique that was the subject of discussion. However, it was not clear that respondents thought their suggestions would be heard. In discussing how law enforcement officers may arrive on scene to support the emergency medical response team, one respondent opined that the decisions about overdose response are made outside the drug using community,

The community does not dictate what the first responders do…. It's beyond us. It's beyond the patient. It's the cops, the paramedic, they have it all worked out. Whatever they do, I don't think any victim or any person that needs an ambulance is going to dictate who shows up and who doesn't. I want the cops. I don't want the cops. It's like, it's—It's what they do. They do it for some reasons.

## Discussion

We conducted three focus groups to elicit the perspectives of PWUDs about a machine-learning technique to identify opioid overdose cases in 911 data and a post-overdose intervention that would be deployed to provide naloxone, training in overdose risk reduction techniques, and/or connection to services like methadone, buprenorphine, or syringe services. This study was motivated by a concern that a well-meaning public health-minded intervention could also produce significant harm to the people it is supposed to benefit. Many technologies are inherently “dual use.” That is, there is a risk that they can be used to design or produce something that causes harm, when causing that harm is not their primary purpose [29]. In this case, a technique to detect opioid overdose emergencies in 911 dispatch data was conceptualized as a way to increase the speed and accuracy with which an intervention could be deployed to the scene. Upon further deliberation, we also wondered whether it could cause harm by putting PWUDs at greater risk for the criminal justice-based consequences they fear. Our findings suggest that participants’ concerns about the technique to detect opioid overdoses and intervention were significant, yet often balanced by the perceived benefits, *but only because they already believe that a law enforcement response will be mobilized*, *no matter what they say or do*. Fears about and the inevitability of a law enforcement response to a 911 call was pervasive in the discussions. So much so, that for many respondents, “calling 911” was synonymous with “calling the cops.” We cannot overstate the significance of this finding. Unlike many members of the general public, who do not often question that a call to 911 will result in a helpful emergency medical response, PWUDs are among those who weigh the risks associated with a call for help and perceive that harmful actions could result [9, 10, 30].

Respondents in this study expressed a desire to seek medical assistance to save the life of someone experiencing an opioid overdose and thought the proposed technique and intervention could help in that effort, which is consistent with a large body of research that demonstrates that PWUD are willing and able to take action in the event of witnessing opioid overdoses [e.g., 14, 31, 32]. Our findings demonstrate that the desire to seek that help is tempered by fears of a potential law enforcement response, which could result in CPS involvement, arrest, incarceration, and threats to personal reputation and privacy. Some respondents mentioned the threat of being charged with murder if they are at the scene of an overdose–a phenomenon that has become more common across the US during this opioid epidemic [33], and is in direct conflict with efforts to engage PWUDs in potentially life-saving intervention efforts. Other concerns related to the machine-learning technique included a loss of autonomy since the caller’s choice not to disclose could be overridden, and a fear that an inaccurate algorithm could result in misdiagnosis and delayed care.

In terms of the intervention model, respondents generally perceived benefit in the deployment of an intervention, especially if the interventionist was a peer, or person with lived experience of substance use, who could be empathetic and provide relevant information and resources. Concerns about the intervention were related to violation of privacy (similar to concerns about identifying opioid overdoses in the 911 data), and a sense that the moments immediately post-overdose are suboptimal for intervention delivery due to the experience of naloxone-precipitated withdrawal symptoms. Referrals alone were seen as insufficient, and more active follow-up and assistance were recommended. Participants emphasized that an intervention should be responsive to their “real problems”, rather than focusing exclusively on substance use disorder treatment linkage. Across all three focus groups, participants expressed a desire for an alternative number that could summon emergency medical assistance without triggering a law enforcement response. However, fundamentally, participants in the current study expressed a sense of helplessness, stemming from the sense that their ideas for how interventions and emergency response systems *should* work would not be heeded, and that as with most drug-related polices in the US, any future interventions would be both designed and implemented from above without their input.

A patient centered perspective on the development of post-overdose interventions could be helpful to inform a more effective model. Patient centered care (PCC) is a perspective that prioritizes attention to the patient’s experience of illness and health care in the design and delivery of high quality care [34]. PCC is guided by 7 dimensions: 1) respect for the patient’s values, preferences, and expressed needs; 2) coordination and integration of care; 3) information, communication, and education; 4) physical comfort; 5) emotional support and alleviation of fear and anxiety; 6) involvement of family and friends; and 7) transition and continuity [35]. In the current study, participants provided perspectives on an ideal intervention that addressed nearly all of these 7 dimensions. Participants expressed preferences and needs related to how an intervention should be delivered (by people with lived experience, at a time that the patient is receptive, in a manner that addresses the patients’ “real needs” and that protects patient privacy and safety). They discussed barriers to access to care for opioid overdose (risks associated with calling 911) and proposed a solution (an alternative number to summon emergency medical assistance), highlighted the value of peer support specialists as empathetic interventionists who can provide support, and described how the intervention could benefit patients *and* their families and friends. The recommendation to provide active follow-up for patients (rather than just referrals) could serve to bolster continuity of care, secure transitions to services, and coordinate care across sectors. Finally, participants discussed timing the intervention to ensure that patients were not physically uncomfortable due to precipitated withdrawal. The only dimension of PCC that was not explicitly discussed by participants in these focus groups was “information and education”, which Gerteis [35] explains is related to patients’ fear that information is being withheld from them during their care. In our study, respondents demonstrated a high level of understanding regarding the mechanisms underlying opioid overdose and the recommended treatment, which affirms previous research demonstrating high levels of knowledge related to opioid overdose among PWUDs [36, 37].

### Limitations

This study should be considered in light of its limitations. Attendance at the focus groups was low, resulting in 3 “mini” focus groups (FG1 n = 2, FG2 n = 6, FG3 n = 3), which is fewer than the recommended 8–10 participants per group. This may have heightened the risk that responses were subject to social desirability bias, especially because focus group-based inquiry is expected to uncover normative beliefs about the topic of study [38] and there could have been greater normative pressure in smaller groups. However, we did observe disagreement among some respondents, suggesting that at least some of them felt comfortable expressing non-normative opinions. Respondents for this study live in a small city in the Western US, therefore their attitudes and beliefs may not be reflective of PWUDs in other communities. Their reports may also reflect a diversity of historical experiences and are not necessarily accounts of recent experiences or experiences in their current city. However, our findings related to concerns about calling 911 are consistent with other studies that used different methods (some conducted in larger urban areas; e.g., [14, 22]), lending support for our conclusions. This study was undertaken to elicit opinions about a proposed intervention that had not yet been implemented. Therefore, the participants had not yet had the opportunity to experience the program, and their opinions may change after exposure to the program. Future research should examine the experiences of and attitudes towards post-overdose intervention programs in the early and later stages of implementation to determine their acceptability among PWUDs.

## Conclusion

The current study builds upon the existing literature by encouraging us to reflect critically on the use of 911 data for the purposes of surveillance and intervention deployment. Interventions that are predicated on engagement with the 911 system for their delivery will only reach a small proportion of the population: those who overdose in the presence of people willing to call 911. For others, including people who use drugs alone and people who use drugs in the presence of individuals who fear calling 911, access to interventions designed to link opioid overdose patients with follow up care and resources will be severely constrained. Relatedly, surveillance efforts that seek to enumerate opioid overdose cases or other types of events based on 911 calls should recognize the inherent limitations of 911 data sets, which consist only of those cases for which a 911 call is made. This may create selection bias by which particularly marginalized or vulnerable individuals are underrepresented, and majority populations are overrepresented. Public health systems must account for the fact that a large share of overdoses will never come to the attention of the emergency response system until fears are mitigated and trust is restored.

In the context of the current North American opioid epidemic, innovative intervention strategies to reduce opioid overdose deaths are urgently needed. To maximize effectiveness, it is imperative to center the perspectives of PWUDs when designing and implementing interventions. In exploring PWUDs’ opinions about a 911-based post-overdose intervention, we found that respondents identified the potentially life-saving nature of a post-overdose intervention. However, fears related to using the 911 system to summon emergency medical assistance were pervasive. Incorporating a patient-centered perspective in intervention design may help improve outcomes and reduce opioid overdose mortality.
